# Experimental and numerical study on cavitation pulsating pressure of water-jet propulsion axial-flow pump

**DOI:** 10.1371/journal.pone.0310167

**Published:** 2024-10-28

**Authors:** Ji-Tao Qiu, Teng-Yan Liu, Xia-Yong Liu, Yuan-Xing Dai, Zong-Long Wang, You-Lin Cai

**Affiliations:** 1 Science and Technology of Water Jet Propulsion Laboratory, Shanghai, China; 2 Marine Design &Research Institute of China, Shanghai, China; Industrial University of Ho Chi Minh City, VIET NAM

## Abstract

Cavitation may occur in water-jet pump during operation of water-jet propulsion vessel, and once cavitation occurs, the tip clearance pulsating pressure of the impeller may be intensified, resulting in increased vibration of the water-jet propulsion unit. In this paper, the cavitation pulsating pressure characteristics at different positions in the pump are studied by experiment and numerical simulation, and the pulsating pressure characteristics in tip clearance are mainly researched. Based on Star-CCM+ commercial software, unsteady Reynolds-averaged Navier-Stokes equations(RANS) numerical simulation is carried out, and the feasibility of the numerical simulation method is verified by uncertainty analysis. The results show that the cavitation pulsating pressure near the leading edge of the impeller in the tip clearance is the largest. The variation of the tip clearance pulsating pressure with the intensification of cavitation is studied by numerical simulation, and its mechanism is revealed. A dimensionless coefficient of net positive suction head (*C*_NPSH_) is proposed, and the study shows that the cavitation pulsation pressure coefficients of pumps of different scales are equal when the working conditions are similar and the *C*_NPSH_ are equal, which indicates that the cavitation pulsating pressure performance of full scale pump can be predicted by model scale. It is of great significance to evaluate the vibration performance of the full scale water-jet propulsion.

## Introduction

Water-jet propulsion has been widely used in high-performance ships and special ships because of its superior maneuverability, strong anti-cavitation ability, less attached body and high propulsion efficiency at high-speed. At present, the matching technology between water-jet propulsion and ship has been very mature, and water-jet propulsion ship generally operates within the cavitation limit line of water-jet propulsion pump. The cavitation limit line of water-jet propulsion pump is obtained according to the net positive suction head (*NPSH*) when the head (*H*) drops 3% due to cavitation deterioration in the model experiment, and at this point, in fact, cavitation has occurred in the pump. Therefore, cavitation may occur on the impeller blade of pump when the water-jet propulsion ship is operating. Cavitation of the impeller of a water-jet propulsion pump usually occurs first near the leading edge of the tip due to the tip leakage vortex. Once cavitation occurs, the tip clearance pulsating pressure may increase rapidly, thus aggravating the vibration of the water-jet propulsion unit. On the other hand, at present, high-performance ships have increasingly higher requirements for the performance of the propulsion pump, therefore it is of great significance to study the cavitation pulsating pressure in the tip clearance of the water-jet propulsion pump.

At present, there are relatively few researches on the tip clearance cavitation pulsating pressure of water-jet propulsion pump. Lu et al. [1] used the unsteady Reynolds-averaged Navier-Stokes equations (RANS) numerical method to conduct numerical simulation of the model pump and compared with the experiment results to verify the reliability of the numerical method, and the maximum error of *H* was less than 10%. Then, taking an underwater vehicle equipped with a water-jet propulsion as the research object, the influence of different size of tip clearance on the surrounding pressure field under cavitation and non-cavitation conditions were studied. Zhao et al. [2] studied the cavitation flow in an axial flow pump by using numerical simulation, and the research focused on the pressure change on the blade surface and the influence of cavitation on the flow state between blades. The Large eddy simulation (LES) or the detached eddy simulation (DES) have also been applied in this research. In theory, these methods are more accurate, but need to pay a greater cost of computing resources. Yang et al. [3] used the DES mothed to analyze the tip clearance pulsating pressure of three axial flow pumps with different load distribution patterns under the condition of non-cavitation, and the study showed that regardless of the load distribution, the pulsating pressure near the leading edge was the largest. Guo et al. [4] carried out a numerical study on the tip clearance pulsating pressure of an axial flow pump, and analyzed the pulsating pressure frequency domain characteristics at different pressure monitoring points under three cavitation conditions. This research showed that the tip clearance pulsating pressure with cavitation was dominated by rotor blade frequency, and the maximum pulsating pressure was at the downstream boundary of the cavitation region. In addition to numerical simulation studies, there are also experimental studies of tip clearance pulsating pressure. Zhang et al. [5] used high-frequency pressure sensors to measure the pulsating pressure at three measuring points of the impeller inlet, impeller outlet and diffuser outlet of a model scale axial-flow pump under three different rotation speed, and analyzed the frequency characteristics of the pulsating pressure, but did not monitor the pulsating pressure in the tip clearance. Shen et al. [6] studied the evolution mechanism of tip cavitation of an axial-flow pump by using high-speed photography, and measured the pulsating pressure in the cavitation region. The results showed that tip cavitation starts from the leading edge and the pulsating pressure at a certain location decreases with the occurrence of cavitation. A large number of studies have been carried out on the cavitation pulsating pressure of centrifugal pumps [7–9], and the research methods used in these researches are similar to those used to study the tip clearance pulsating pressure of axial flow pumps. But the centrifugal pump is not used for water-jet propulsion, and the research shows that its internal flow field, cavitation characteristics are very different from the axial-flow pump [10, 11].

In addition, Dong et al. [12] carried out an experimental study on the tip clearance pulsating pressure of the tunnel thruster propeller, and investigated the influence of flow velocity, rotation speed and cavitation on the pulsating pressure. Experimental results indicated that influence of flow and rotation speed on the pulsating pressure was negligible, and the pulsating pressure increased as the cavitation number decreases. Shu et al. [13] conducted an experimental study on the tip clearance pulsating pressure of the ducted propeller, and obtained the circumferential and axial distribution of the pulsating pressure along the inner wall of the duct. The results showed that the pulsating pressure of the duct was dominated by first-order blade frequency, and the pulsating pressure amplitude at different circumferential measuring points was similar. Tip clearance exists in both the tunnel thruster and the ducted propeller, which is similar to that of the water-jet propulsion axial-flow pump, but the water-jet propulsion pump has the characteristics of smaller tip clearance, longer tip chord and stronger work ability of the blade tip. Therefore, the clearance flow is more complicated, and the influence of pulsating pressure is often more significant.

In general, it is still necessary to carry out detailed analysis and research on the tip clearance pulsating pressure of water-jet propulsion pump under cavitation conditions. Especially for the problem of how to predict the performance of tip clearance cavitation pulsation pressure of full scale water-jet propulsion pump by studying the model scale. In this paper, the tip clearance pulsating pressure experiment and unsteady RANS numerical simulation of a water-jet propulsion axial-flow pump under cavitation conditions are carried out. The uncertainty of the numerical simulation in this paper is evaluated, and the numerical simulation method of tip clearance cavitation pulsating pressure is established, which can provide a numerical method for evaluating tip clearance cavitation pulsating pressure. Based on this numerical method, the distribution of the pulsating pressure on the inner surface of the axial-flow pump shroud is investigated, and the variation of the pulsating pressure in the tip clearance of the impeller under different cavitation conditions is mainly analyzed. The variation mechanism of the tip clearance pulsating pressure is revealed by numerical analysis. Finally, the tip clearance pulsating pressure characteristics of different scale pumps are preliminarily studied, which can be used to predict the tip clearance pulsating pressure of full scale water-jet propulsion pump by model scale performance.

## Research object and method

### Axial-flow pump geometry

**Fig 1:**
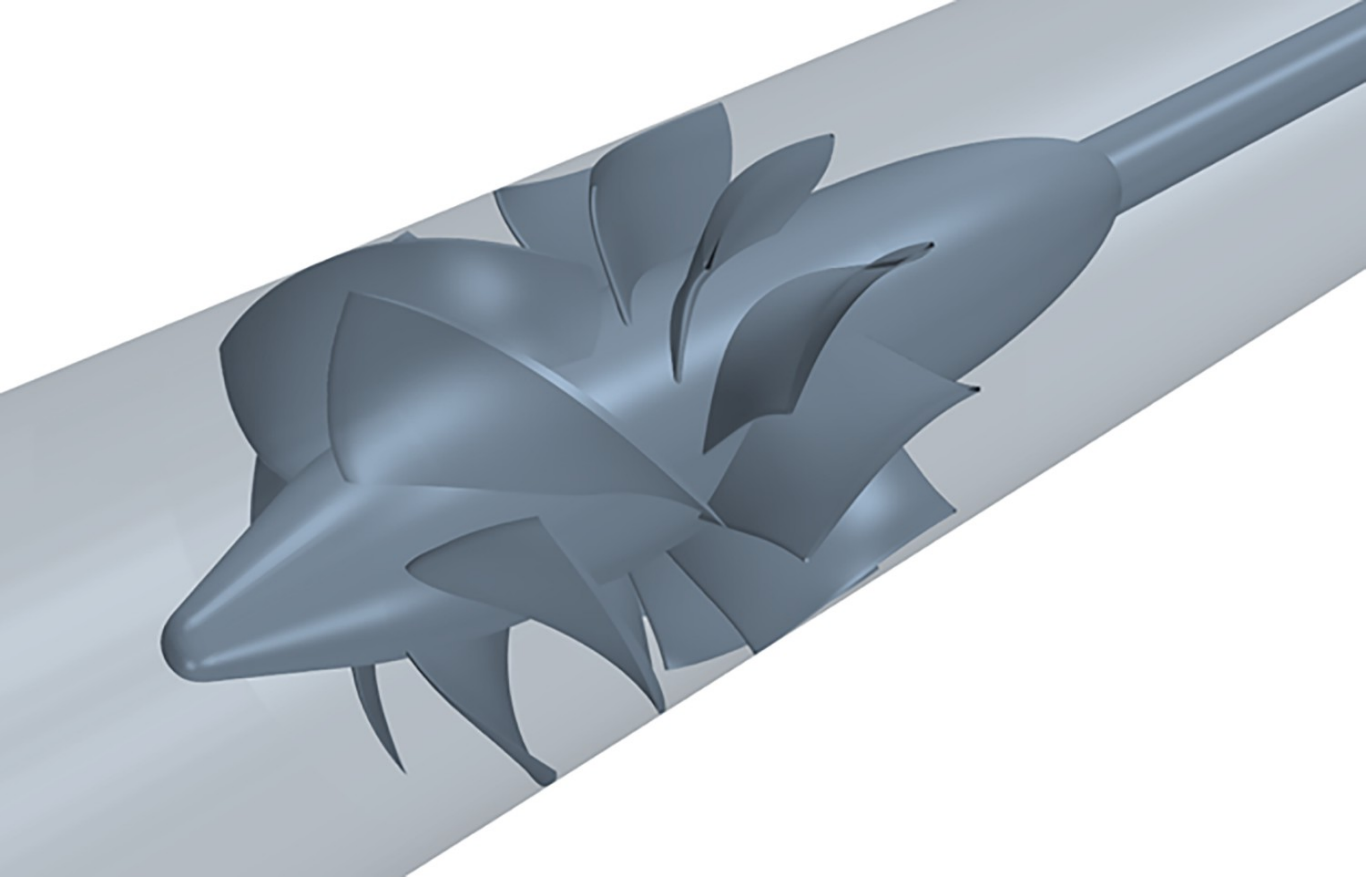
Axial flow pump model.

In this paper, a water-jet propulsion axial-flow pump is taken as the research object. The axial-flow pump consists of a 6-blade impeller and an 11-blade diffuser, and the axial flow pump model is shown in Fig 1. The model diameter of the axial-flow pump is *D* = 0.3m, and the tip clearance of the impeller is 0.001*D*.

### Numerical simulation method

**Fig 2:**
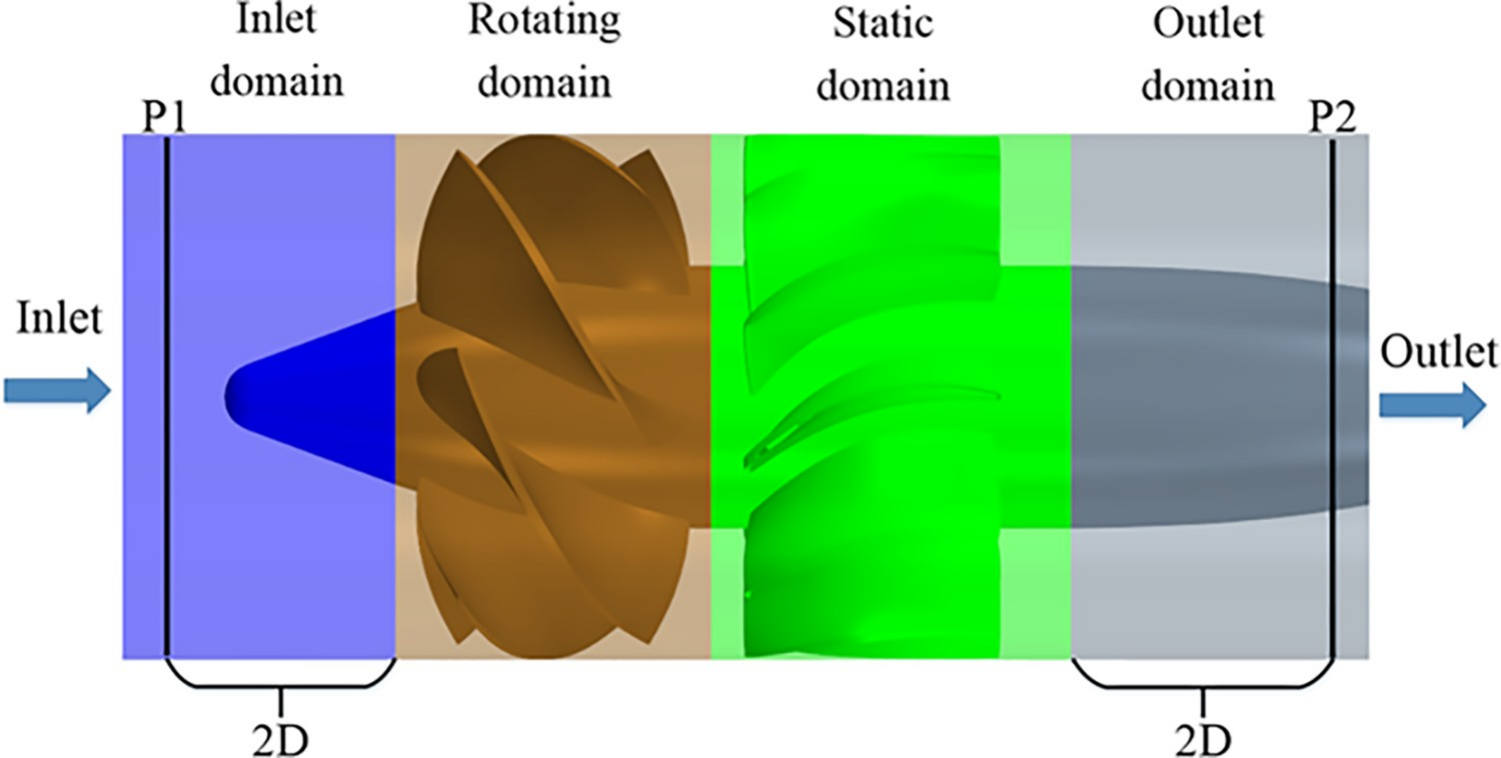
Computational domain.

The numerical simulation in this study is based on Star-CCM+ commercial software. The computational domain is a cylinder and is divided into 4 subdomains, including the inlet domain, the rotating domain containing the impeller, the static domain containing the diffuser, and the outlet domain. The computational domain is shown in Fig 2. The upstream boundary of the inlet domain is set to the volume flow inlet, and the downstream boundary of the outlet domain is set to the pressure outlet. At the inlet and the outlet, the turbulence intensity and the turbulent viscosity ratio are set to 2% and 2, respectively. The numerical simulation data is transmitted between subdomains through the Interface. In this study, the computational domain is discretized into block-structured hexahedral cells generated by ANSYS ICEM commercial software. The grid generation method is the same as that used in [14].

Zhang et al. [15] carried out a RANS numerical simulation and experimental study of propeller cavitation using Star-CCM+ commercial software. The Schnerr-Sauer cavitation model was used in their numerical simulation, and the simulation results of realizable *k-ε* and SST *k-ω* turbulence models were compared. Their results showed that the results of the two turbulence models are similar and in good agreement with the experiments. Therefore, the unsteady RANS method is used for numerical simulation, and the two-layer realizable *k*-*ε* model is used to closure the governing equations in this paper. The two-layer approach was first proposed by Rodi [16]. As another method of applying the *k-ε* model in the viscous sub-layer and the buffer layer, it works with either low-Reynolds number type meshes (*y*^+^ ~1) or wall-function type meshes (*y*^+^ > 30). The governing equations are discretized with second-order schemes both in space and time, and solved by the semi-implicit method for pressure-linked equations (SIMPLE). The Schnerr-Sauer cavitation model is widely used in rotating machinery and has a good effect on the simulation of cavitation [17], thus it is used to simulate cavitation in this paper. By changing the pressure value at the pressure outlet, the environmental pressure can be changed to simulate different cavitation conditions. In the numerical simulation, the reference pressure is set to 0 Pa. The rotation speed *N*_R_ of the impeller is set to 1450 r/min, which is consistent with the rotation speed in the experiment.

The moving reference frames (MRF) method is used for steady numerical simulation, and the result of steady numerical simulation is taken as the initial value for unsteady numerical simulation to stabilize and accelerate the convergence of unsteady numerical simulation. For unsteady numerical simulation, the criterion of simulation convergence is that the time average of *H* changes within ±0.01% in each of the 5 consecutive rotation periods (impeller rotate 5 revolutions). The definition of *H* is consistent with the definition in the experiment and is defined as

$$H=\frac{p_{2}-p_{1}}{\rho g}+\frac{V_{2}^{2}-V_{1}^{2}}{2g}$$ (1)

where *p*_1_ and *p*_2_ denote the average pressure over the circumference near the inner wall of the pump shroud at position *P1* and *P2*, respectively (pa); *V*_1_ and *V*_2_ denote the average axial velocities of cross sections at position *P1* and *P2*, respectively (m/s); *ρ* denotes the density of water (kg/m^3^); *g* denotes the gravitational acceleration (m/s^2^). The positions of *P1* and *P2* are shown in Fig 2 and are consistent with the experiment.

### Experiment method

**Fig 3:**
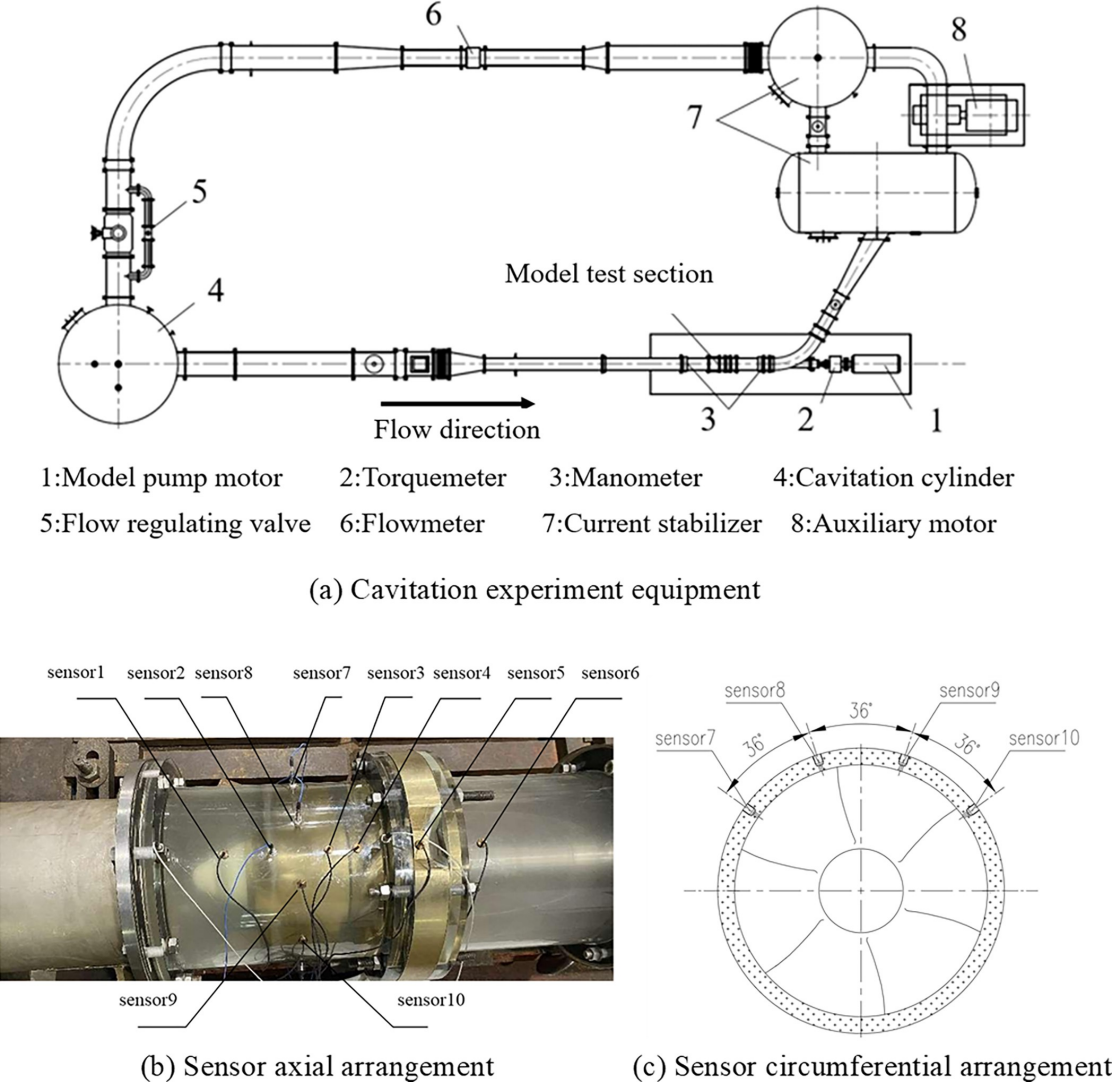
Pulsating pressure sensor arrangement and cavitation experiment equipment.

The cavitation pulsating pressure experiment of water-jet propulsion pump is carried out in the Science and Technology of Water Jet Propulsion Laboratory. The pulsating pressure sensor arrangement and cavitation experiment equipment are shown in Fig 3. The cavitation experiment equipment is shown in Fig 3(A). The pulsating pressure on the inner wall of the pump shroud is measured by using a series of pulsating pressure sensors inserted into the surface of the pump shroud, and the position of the sensors is shown in Fig 3(B) and Fig 3(C). Sensor1 is located in the upstream of impeller, sensor2 is located near the leading edge of the impeller, sensor3 is located near the trailing edge of the impeller, sensor4 is located between the impeller and the diffuser, sensor5 is located in the middle of the chord of the diffuser and is located in the middle of two adjacent diffuser blades, sensor6 is located in the downstream of the diffuser. Sensor7~10 are located in the middle of the chord of the impeller, and their circumferential arrangement is shown in Fig 3 (B). Sensors 2,3,7 ~10 are both located in the tip clearance. Sensor 2 has a range of 0–3200 kPa and the remaining sensors have a range of 0–800 kPa.

In the experiment, the rotation speed of the propulsion pump is 1450 r/min, and the flow rate *Q* = 0.46 m^3^/s (Design condition). The sampling frequency is 6400Hz. The pressure in the experiment device is gradually reduced by the decompression device to realize the pulsating pressure test under different cavitation condition (different *NPSH* values). *NPSH* is defined as

$$NPSH=\frac{p_{1}-p_{V}}{\rho g}+\frac{V_{1}^{2}}{2g}$$ (2)

where *p*_v_ is the saturated vapor pressure of water, the value is the saturated vapor pressure of water at a temperature of 16.7°C, and its value in numerical simulation is consistent with the experiment. The *NPSH* value, where *H* decreases by 3% as the *NPSH* decreases, is defined as the requisite net positive suction head (*NPSH*_R_). *NPSH*_R_ is usually obtained by interpolating the *H*-*NPSH* curve obtained by experiments. The *NPSH*_R_ under different operating conditions constitutes the cavitation limit line used in engineering.

## Numerical uncertainty analysis

In order to verify the accuracy of the numerical simulation method in this paper, the uncertainty analysis of the unsteady numerical simulation is carried out using the method recommend by ITTC [18] and adopted in [19]. The uncertainty analysis consists of two processes: verification and validation.

### Verification

**Fig 4:**
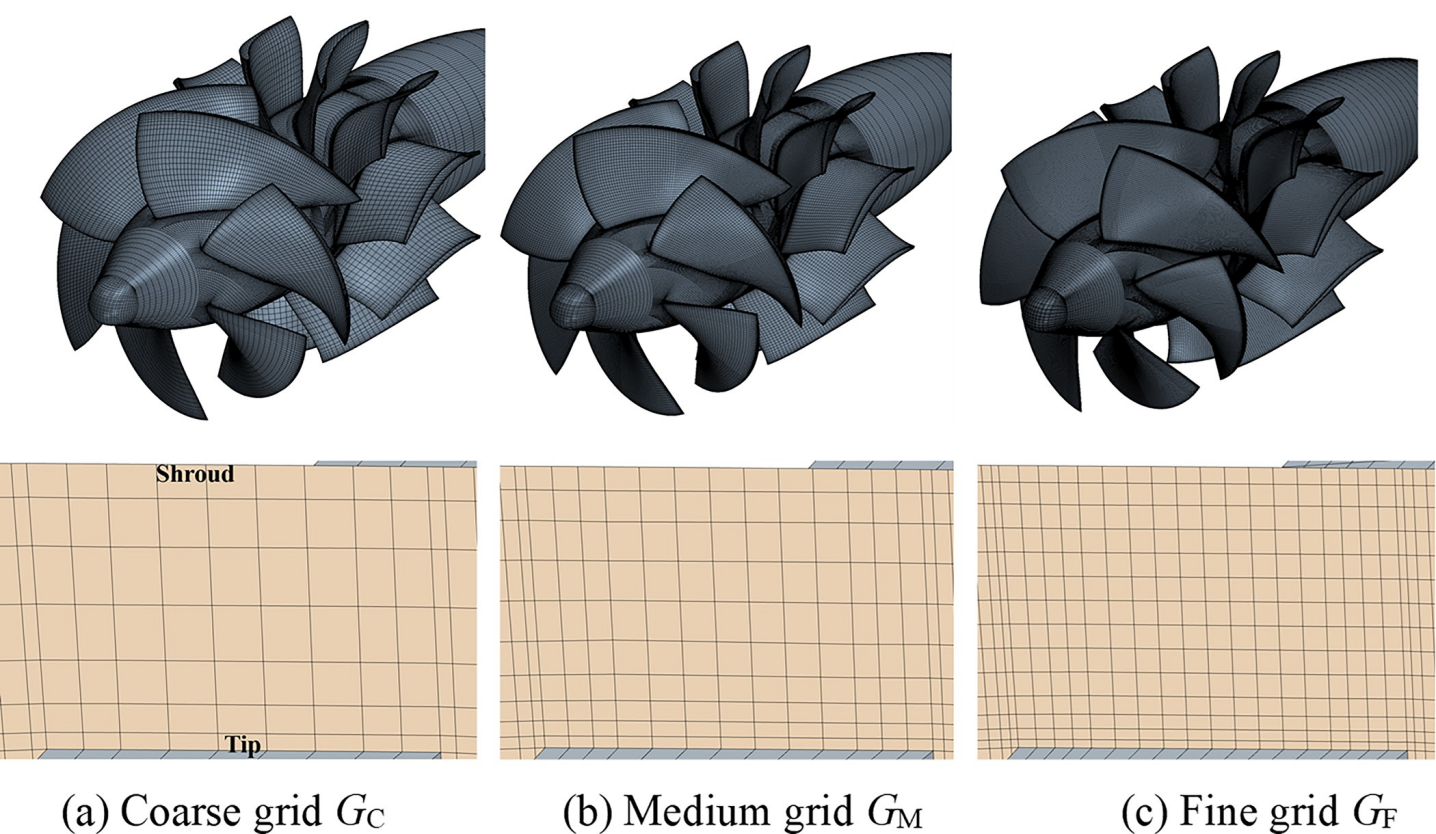
The Grids on the surface (Top row) and in tip clearance (Bottom row).

According to the requirements of uncertainty analysis, three sets of successively refined grid with uniform grid refinement ratio ($r_{G}=\sqrt{2}$) are generated, namely, coarse grid (*G*_C_), medium grid (*G*_M_) and fine grid (*G*_F_), and the grids on the surface (top row) and in tip clearance (bottom row) are shown in Fig 4.

In fact, parameters in turbulence models and cavitation models can be classified as other parameters for uncertainty analysis according to the procedures recommended by ITTC [18]. There are many parameters in turbulence models and cavitation models, and the workload is very large if the uncertainty analysis of these parameters is carried out. As mentioned above, the k-ε turbulence model and the Schnerr-Sauer cavitation model are used to simulate cavitation, which can meet the operational requirements in most condition. The research conducted by Gaggero et al. [20] and Sajedi et al. [21] further validated this approach. Therefore, only the uncertainties caused by grid size and time step size are analyzed in this study. According to the unsteady numerical uncertainty analysis method, the simulation numerical uncertainty (*U*_SN_) can be defined as,

$$U_{SN}^{2}=U_{I}^{2}+U_{GT}^{2}$$ (3)

where *U*_I_ denotes the uncertainty caused by iteration, *U*_GT_ denotes the uncertainty caused by the grid size and time step size. The time step and the grid size must change simultaneously by using this method to solve numerical uncertainty. Since $r_{G}=\sqrt{2}$ is used in this paper, and the governing equations are discretized with second-order scheme both in space and time, the time refinement ratio is also $r_{T}=\sqrt{2}$ in order to ensure consistent error accuracy. The key parameters of the grid and time step used in this study are shown in Table 1. Corresponding to the height of the first layer in Table 1, the y^+^ value is about 1, to resolve the flow in viscous sub-layer. The time step corresponding to the *G*_F_ is the time taken by the impeller to rotate one degree, i.e., about 1.149425×10^-4^s.

**Table 1:** Key parameters of the grid and time step.

Grid	Maximum Cell Size at Blade Surface (mm)		First-Layer Cell Height at Blade Surface (mm)		Number of Cells in Tip Clearance		Total Number of Cells (Million)	Time Step (deg/step)
	impeller	diffuser	impeller	diffuser	Radial	Circumferential		
*G*_C_	4	5	0.02	0.2	7	9	2.023	2
*G*_M_	2.8	3.5	0.014	0.14	10	13	5.512	1.4142
*G*_F_	2	2.5	0.01	0.1	14	18	15.776	1

The uncertainty analysis is carried out for the numerical simulation of three different *NPSH* conditions, namely, *NPSH* = 6.27m, 10.15m and 12.42m. The numerical simulation results and experiment data (*D*_E_) of *H* are shown in Table 2. According to Table 2, the numerical simulation results of *NPSH* = 10.15 and 12.42m converge monotonically, and the numerical simulation results of *NPSH* = 6.27m converge oscillatively.

**Table 2:** Numerical simulation and experiment data of *H* with different *NPSH*.

*NPSH* (m)	*H*(m)			
	*G*_C_	*G*_M_	*G*_F_	*D*_E_
6.27	12.15	12.68	12.34	11.9
10.15	13.22	13.14	13.09	12.65
12.42	13.27	13.18	13.13	12.73

The calculation process of *U*_GT_ and *U*_I_ for *H* is referred to [18] and [19]. In order to calculate the oscillation correctly caused by numerical iteration, the pulsation caused by physics factors (for instance, the blade frequency component of the pulsation) needs to be removed from the numerical simulation results by filtering. The calculation results of *U*_I_ and *U*_GT_ are shown in Table 3, where the percentages of results are based on the numerical results of grid *G*_F_ in Table 2. Table 3 shows that *U*_I_ is more than one order of magnitude smaller than *U*_GT_, which indicates that iteration has little impact on numerical results. Therefore, it is considered that the *U*_GT_ is available, and *U*_I_ can be ignored, then *U*_SN_≈*U*_GT_. According to the results of *U*_GT_ listed in Table 3, the numerical uncertainty caused by grid and time step in numerical simulation is about 2.1%.

**Table 3:** Calculation results of *U*_I_ and *U*_GT_.

*NPSH* (m)	*U*_I_ (%)			*U*_GT_ (%)
	*G*_C_	*G*_M_	*G*_F_	
6.27	0.14	0.13	0.1	2.1
10.15	0.06	0.05	0.05	1.1
12.42	0.05	0.03	0.02	0.67

### Validation

Validation is the process of analyzing errors in numerical modeling, such as cavitation models. The comparison error *E* is defined as the difference between the experiment data (*D*_E_) and the simulation data (*D*_S_),

$$E=D_{E}-D_{S}$$ (4)

The validation uncertainty (*U*_V_) is defined as

$$U_{V}^{2}=U_{D}^{2}+U_{SN}^{2}$$ (5)

where *U*_D_ denotes experimental uncertainty, *U*_D_ = 2%*D*_E_ in this paper. To determine whether the validation has been achieved, the absolute value of *E* is compared to the validation uncertainty (*U*_V_) and the programmatic validation requirement (*U*_reqd_), and *U*_reqd_ = 5%*D*_E_ in this paper. If the three variables are unequal to each other, one of the following cases must be true,

        1) $|E|<U_{V}<U_{reqd}$                4) $U_{V}<|E|<U_{reqd}$

        2)$|E|<U_{reqd}<U_{V}$                5) $U_{V}<U_{reqd}<|E|$

        3)$U_{reqd}<|E|<U_{V}$                6) $U_{reqd}<U_{V}<|E|$

In the first three cases, the validation is achieved at the *U*_V_ level. Particularly, in the first case the validation is successfully achieved at a level below *U*_reqd_. In the last three cases, the modeling error can be estimated. If |*E*| >> *U*_V_, the modeling error dominates and is approximately equal to *E*. In case 4), the validation is successful at the |*E*| level below *U*_reqd_.

The |*E*| can be obtained according to Eq (4), and the *U*_V_ can be obtained according to Eq (5). Their results are listed in Table 4, where the percentages of results are based on the *D*_E_, and show that the validation of the three *NPSH* conditions is achieved at the |*E*| level, and |*E*| is smaller than *U*_rqed_. It can be concluded that the numerical simulation method adopted in this paper is feasible, and the error is mainly caused by modeling error, such as turbulence model and cavitation model. In the following unsteady numerical simulations, fine grids and corresponding time steps are used.

**Table 4:** Results of |*E*| and *U*_V_.

*NPSH*(m)	|*E*| (%)	*U*_V_ (%)
6.27	3.7	3.0
10.15	3.5	2.3
12.42	3.1	2.1

## Results and analysis

**Fig 5:**
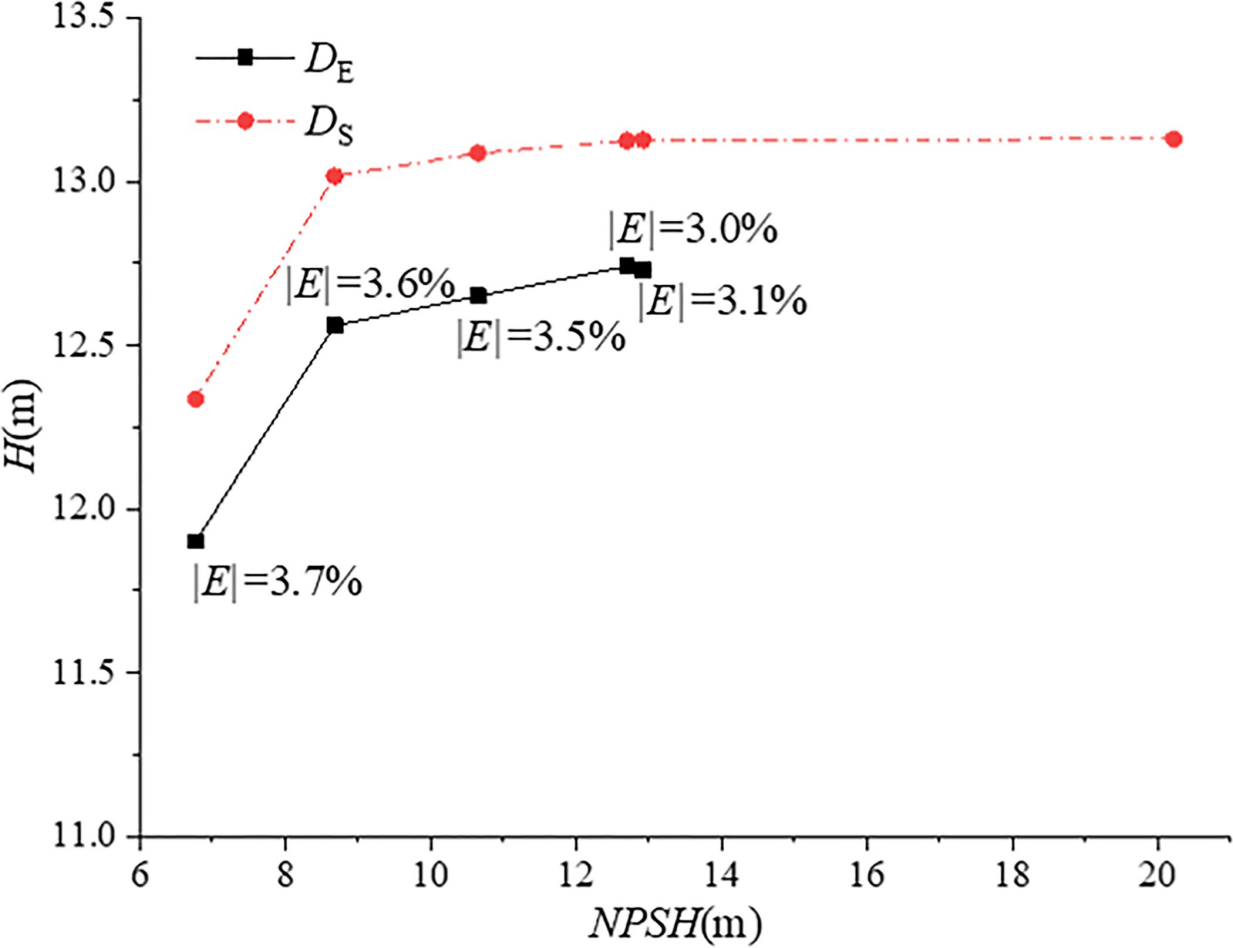
Comparison of *H* between simulation and experiment.

In order to study the tip clearance pulsation pressure characteristics under different cavitation conditions, experimental tests and unsteady numerical simulations of the water-jet pump with different *NPSH* values are carried out. By adjusting the pressure at the pressure outlet, the difference of pressure *p*_1_at *P1* between the numerical simulation and the experiment is within 50Pa, in order to simulate the experiment accurately. According to Eq (1) and Eq (2), if the pressure at *P1* of the experiment and the numerical simulation is approximately equal, the error of *H* is mainly determined by the simulated pressure *p*_2_ at *P2*. Therefore, the similarity of the pressure in the pump is verified by comparing the *H* data of the experiment and the numerical simulation, and the results of comparison of *H* between simulation and experiment are shown in Fig 5. The shape of the *H*-*NPSH* curve obtained by the experiment is similar to that in [2]. According to Fig 5, in the range of *NPSH* values tested, the shape of the simulated *H*-*NPSH* curve is similar to that obtained in the experiment, but the numerical results are about 3% higher than the experiment results, indicating that the simulated pressure difference between the *p*_1_ and *p*_2_ of the pump is close to the experiment value. Limited by the ability to increase pressure of the experiment device, the maximum *NPSH* in the experiment is 12.42m, and cavitation has occurred in this condition (show in Fig 8(A)). However, the numerical results show that *H* is almost constant with pressure increasing. At *NPSH* = 6.27m, the *H* decreases by more than 3%, and the *NPSH*_R_ is between *NPSH* = 6.27m and *NPSH* = 8.18m.

**Fig 6:**
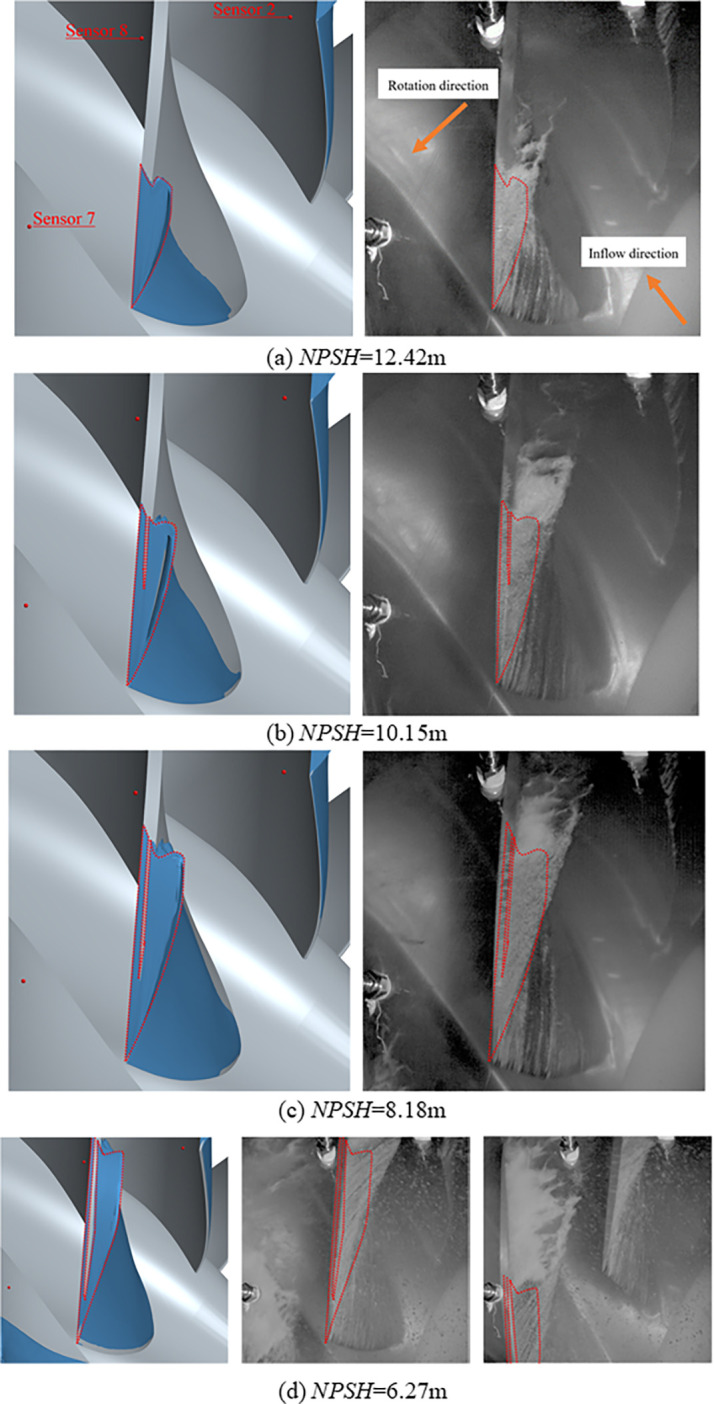
Comparison of cavitation shape between numerical simulation (Left column) and experiment (Right column).

The tip clearance pulsating pressure is closely related to the tip clearance cavitation shape. Therefore, the tip clearance cavitation shape captured in the experiment is compared with that in the numerical simulation, with a volume fraction of 0.5 in numerical simulation. The comparison of cavitation shape between numerical simulation (left column) and experiment (right column) is shown in Fig 6. Both numerical simulation and experiments show that tip clearance cavitation starts from the leading edge, develops along the chord and towards the suction side with the decrease of *NPSH*, and there is also a large area of cavitation on the suction surface. The experiment shows that a stable cavitation region like a triangle is formed on suction side, and it breaks at a certain position, resulting in unstable small bubbles. These bubbles form an unstable cavitation region. In order to show the difference between the cavitation shapes of the numerical simulation and the experiment, the composition of the numerical simulation and the experiment photos is almost the same. The numerical simulation can only simulate the stable cavitation region(as marked by the dotted line in left column in Fig 6, and the dotted line in right column is the same as it), whose shape is similar to that in the experiment, but it cannot simulate the phenomenon of small bubbles generated by cavitation breakage.

**Fig 7:**
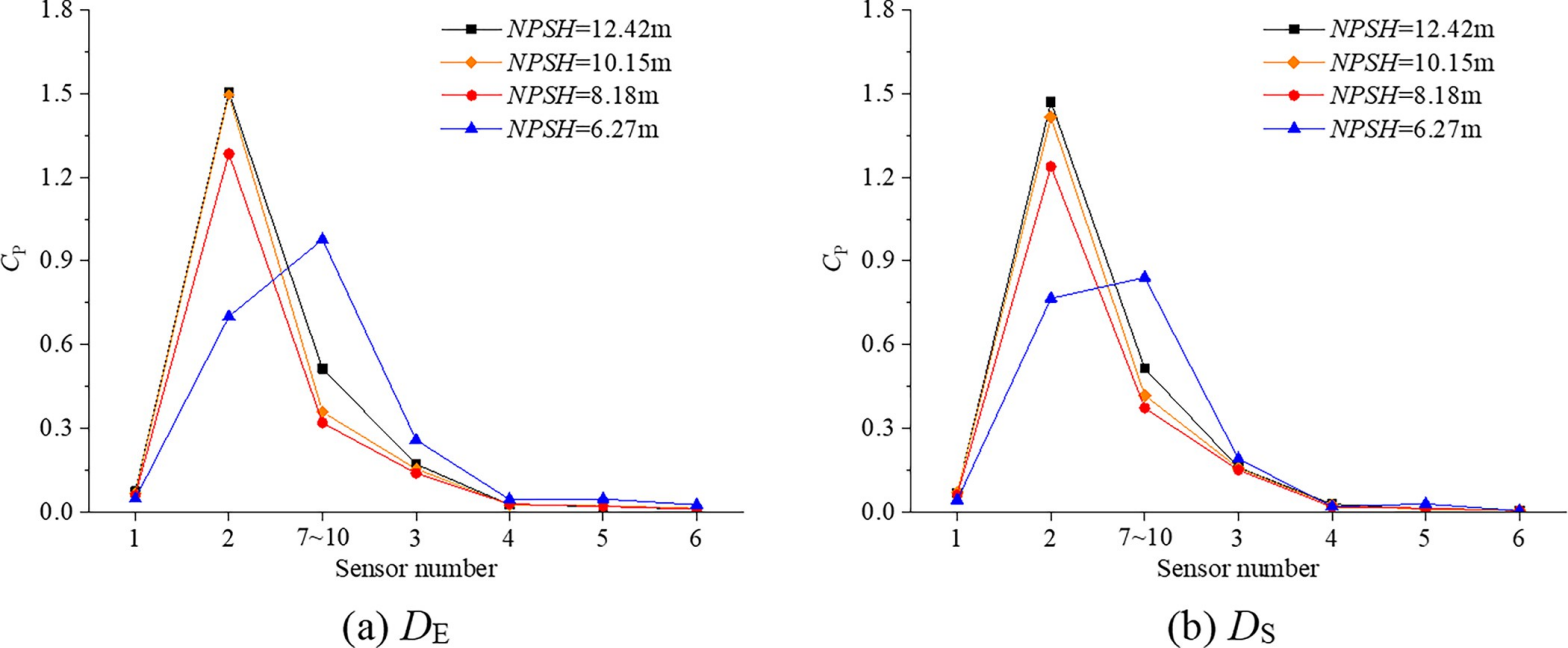
Root mean square (*RMS*_10_) of the blade frequency component (first 10 orders only) at different axial positions.

In this paper, the pressure is converted into the pressure coefficient (*C*_P_), which is defined as,

$$C_{p}=\frac{p}{\rho {\left(N_{R}/60\right)}^{2}D^{2}}$$ (6)

where *p* is the pressure value obtained by numerical simulation or experiment. The pressure coefficient *C*_P_ is transformed by fast Fourier transform, and the root mean square (*RMS*_10_) of the blade frequency component (first 10 orders only) of pulsating pressure at different axial positions are investigated. The *RMS*_10_ value is used to characterize the total energy of the pulsating pressure at a monitoring point. The data at sensor 7~10 is averaged as the data at one location (middle of chord). The results of root mean square (*RMS*_10_) of the blade frequency component (first 10 orders only) at different axial positions are shown in Fig 7, which indicates the axial variation of the RMS_10_ in the range from the upstream of the impeller to the downstream of the diffuser. With the intensification of cavitation, the *RMS*_10_ near the leading edge (sensor2) gradually decreases, and the decreasing speed increases. The *RMS*_10_ at the middle of the chord (sensor 7~10) decreases first, and then increases rapidly at *H* = 6.27m, while the cavitation at the middle of the chord is breaking (show in Fig 6(D)). Fig 7 also shows that the pulsating pressure in the tip clearance is much larger than the pulsating pressure outside the tip clearance, and the pulsating pressure outside the tip clearance is small. Therefore, only the cavitation pulsation pressure in the tip clearance is studied in the following sections.

Table 5 shows the comparison of *RMS*_10_ of sensor2, sensor7~10 and sensor3 in the tip clearance between *D*_E_ and *D*_S_, and most of the errors are within 20%. By comparing Fig 6 and Table 5, it can be found that when the monitoring point is located in the stable cavitation region or has not been affected by cavitation, the error of the numerical simulation is less than 10%. When the monitoring point is located at the boundary of the stable cavitation region or in the unstable cavitation region, the numerical simulation cannot simulate the unstable cavitation region formed by the bubble breaking, therefore the error of the simulation is large, such as *NPSH* = 10.15 and *NPSH* = 8.18m at sensor7~10; *NPSH* = 6.27m at sensor3.

**Table 5:** Comparison of *RMS*_10_ in the tip clearance.

*NPSH* (m)	Sensor Number	*RMS*_10_		Error (%)
		*D*_E_	*D*_S_	
12.42	2	1.504	1.470	-2.2
	7~10	0.514	0.514	0.2
	3	0.171	0.159	-7.1
10.15	2	1.496	1.416	-5.3
	7~10	0.358	0.419	16.8
	3	0.154	0.157	1.8
8.18	2	1.284	1.239	-3.5
	7~10	0.319	0.373	16.7
	3	0.138	0.150	8.7
6.27	2	0.701	0.766	9.3
	7~10	0.977	0.841	-13.9
	3	0.258	0.190	-26.4

**Fig 8:**
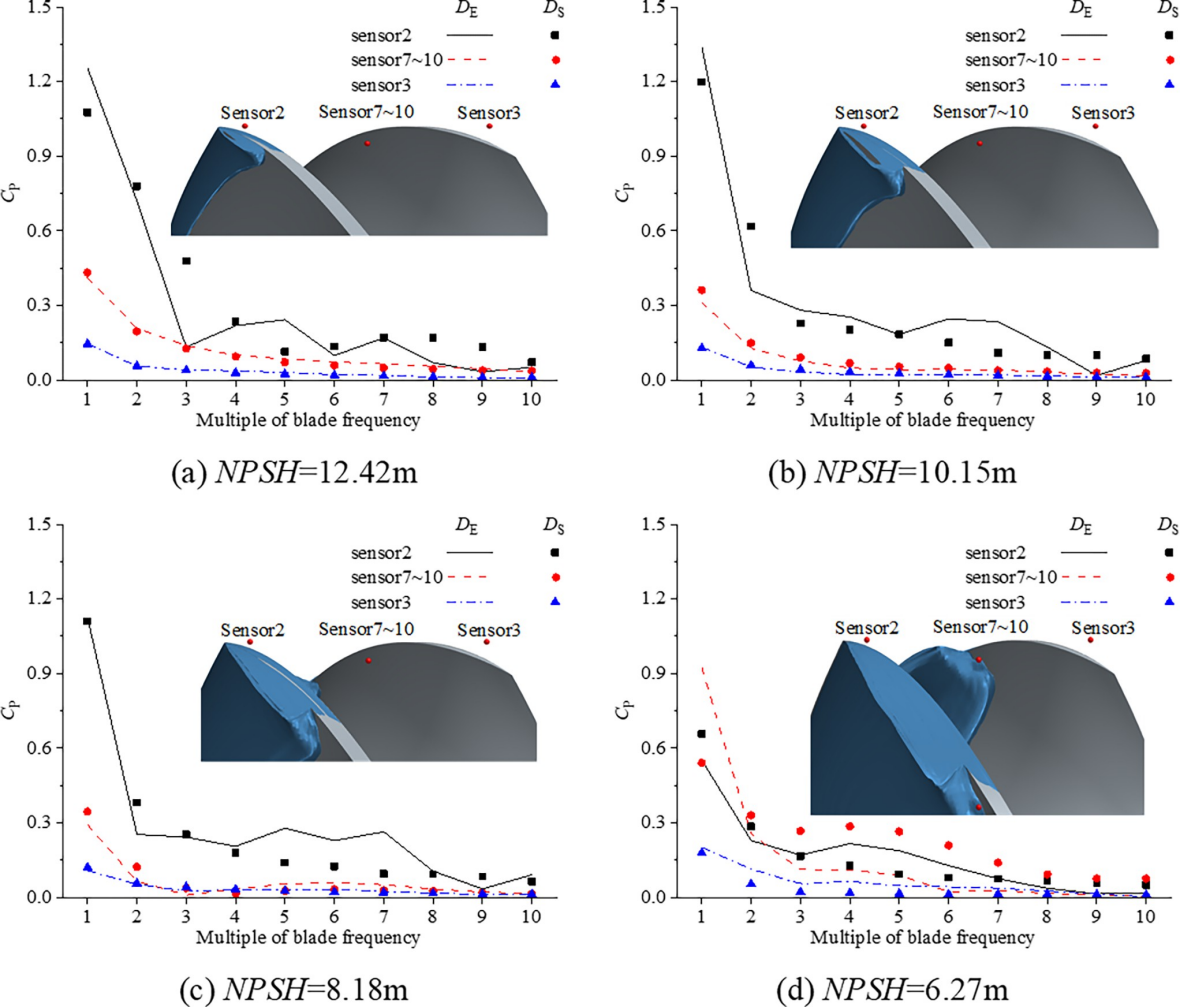
First 10 blade frequency components of pulsating pressure in the tip clearance with different *NPSH*.

Fig 8 shows the first 10 blade frequency components of pulsating pressure in the tip clearance with different *NPSH* at three pressure monitoring points. The axial relative positions of the cavitation area and the monitoring points are also shown in Fig 8. Both experiments and numerical simulations show that the first order blade frequency component of cavitation pulsating pressure at three different monitoring points is the largest, and the pulsation pressure decreases with the increase of frequency. In addition, although the simulation error of *RMS*_10_ is small in some conditions, there are still large errors in the simulation of each order frequency component of pulsating pressure, especially there is cavitation, such as the pressure at sensor2 shown in Fig 8. For the severe cavitation condition, the numerical simulation error is large regardless of *RMS*_10_ or each order blade frequency component, as shown in Fig 8(D).

**Fig 9:**
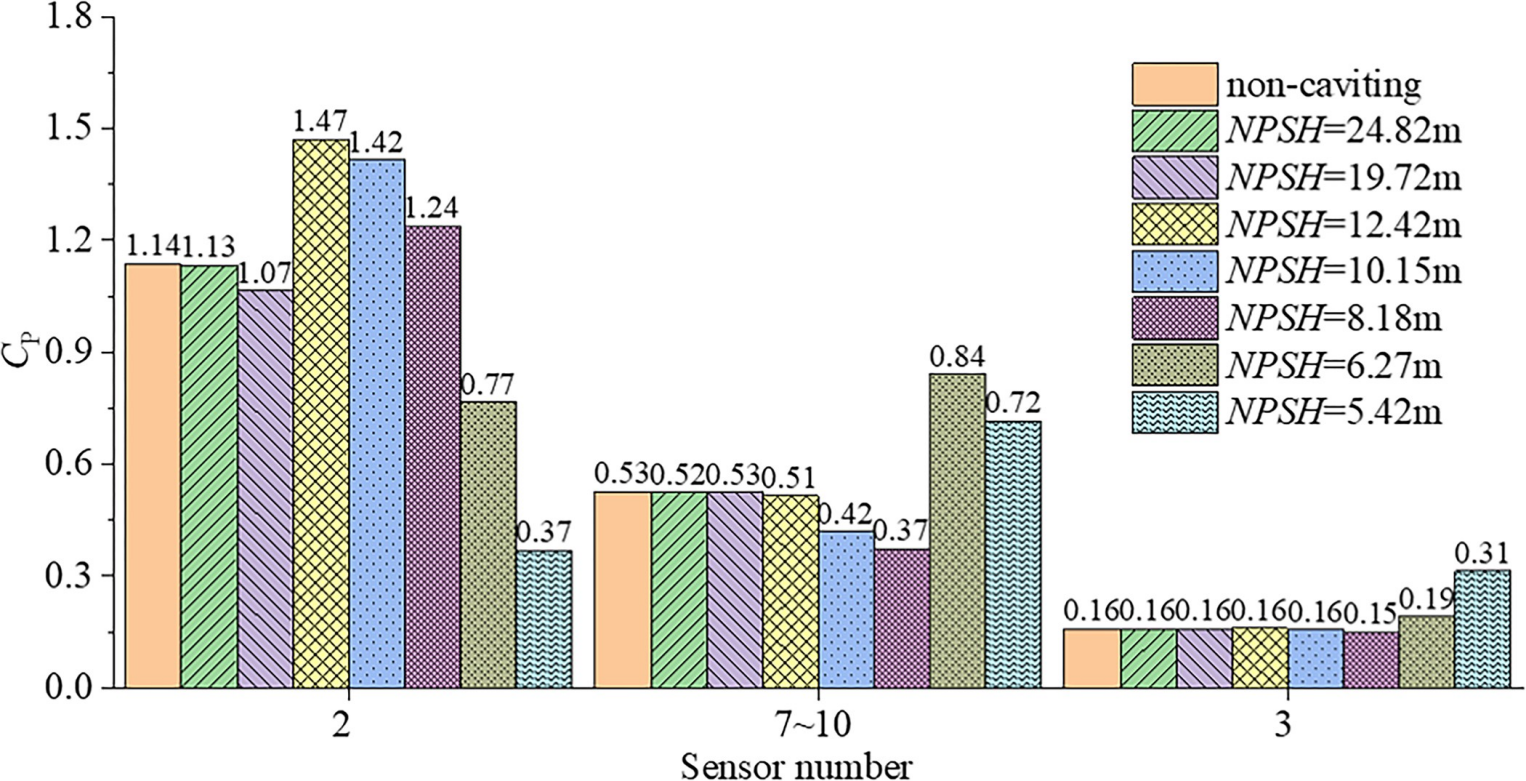
Variation of *RMS*_10_ of tip clearance pulsating pressure with the intensification of cavitation.

Fig 9 shows the variation of *RMS*_10_ of tip clearance pulsating pressure with the intensification of cavitation, from non-cavitating to *NPSH* = 5.42m. The pulsating pressure at sensor2 and sensor7~10 decrease first, then increase, and then decrease with the intensification of cavitation. The pulsating pressure at sensor3 decreases first with the intensification of cavitation, and then gradually increases when *NPSH* = 6.27m. The results in Fig 9 show that there is a rule that the pulsating pressure at a certain point in the tip decreases first, then increases, and then decreases with the gradual deterioration of cavitation, and also shows the maximum *RMS*_10_ of pulsating pressure in the tip clearance is still near the leading edge when cavitation occurs, although the most severe cavitation is not near the leading side. The maximum *RMS*_10_ of pulsating pressure near the leading edge without cavitation is 1.14 and increases to 1.47 due to cavitation, about 1.3 times. The maximum *RMS*_10_ of pulsating pressure near the middle of chord increases from 0.65 to 1.05, about 1.62 times. The increment of pulsating pressure due to cavitation should be different for completely different pumps.

**Fig 10:**
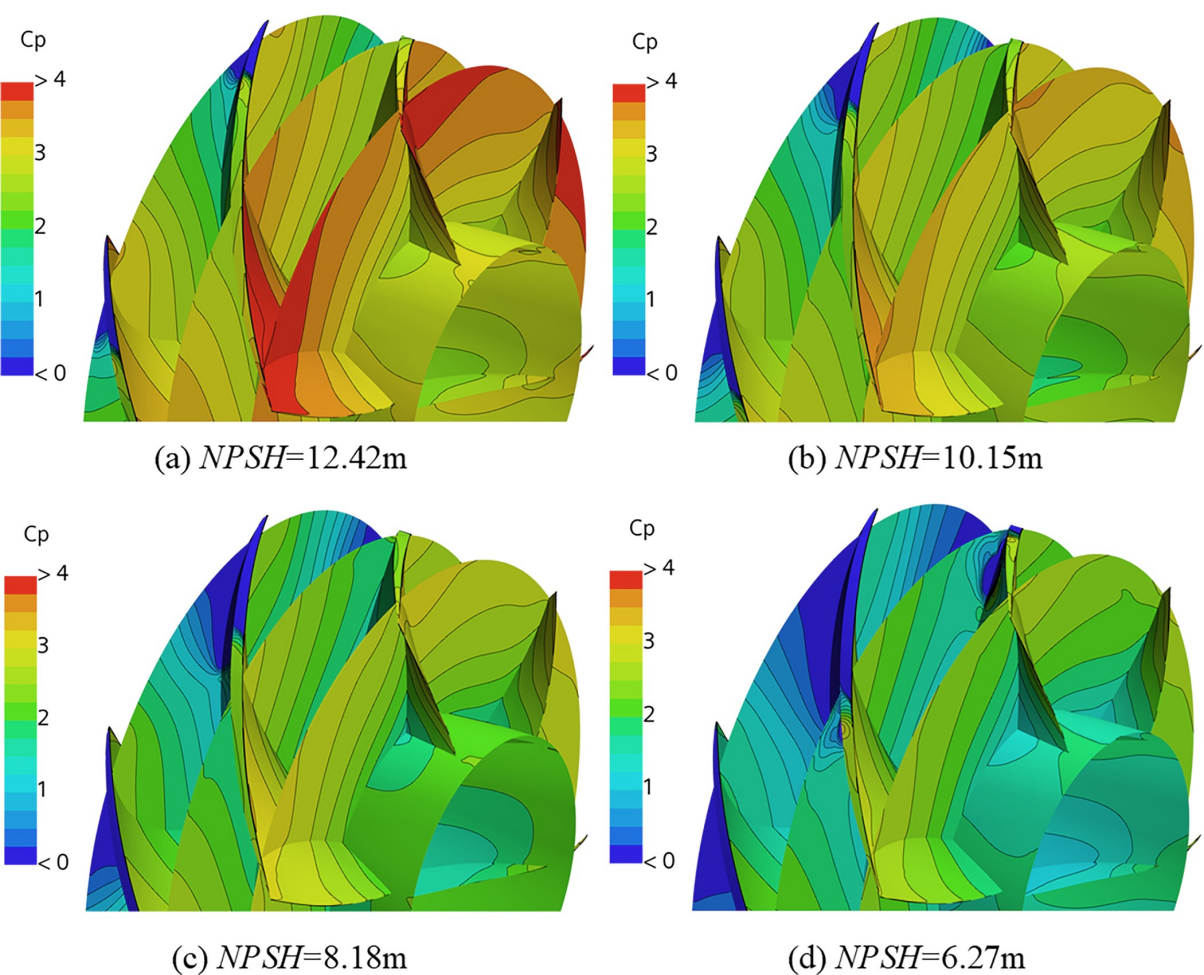
Distribution of pressure coefficient on three cross sections and blade surfaces.

**Fig 11:**
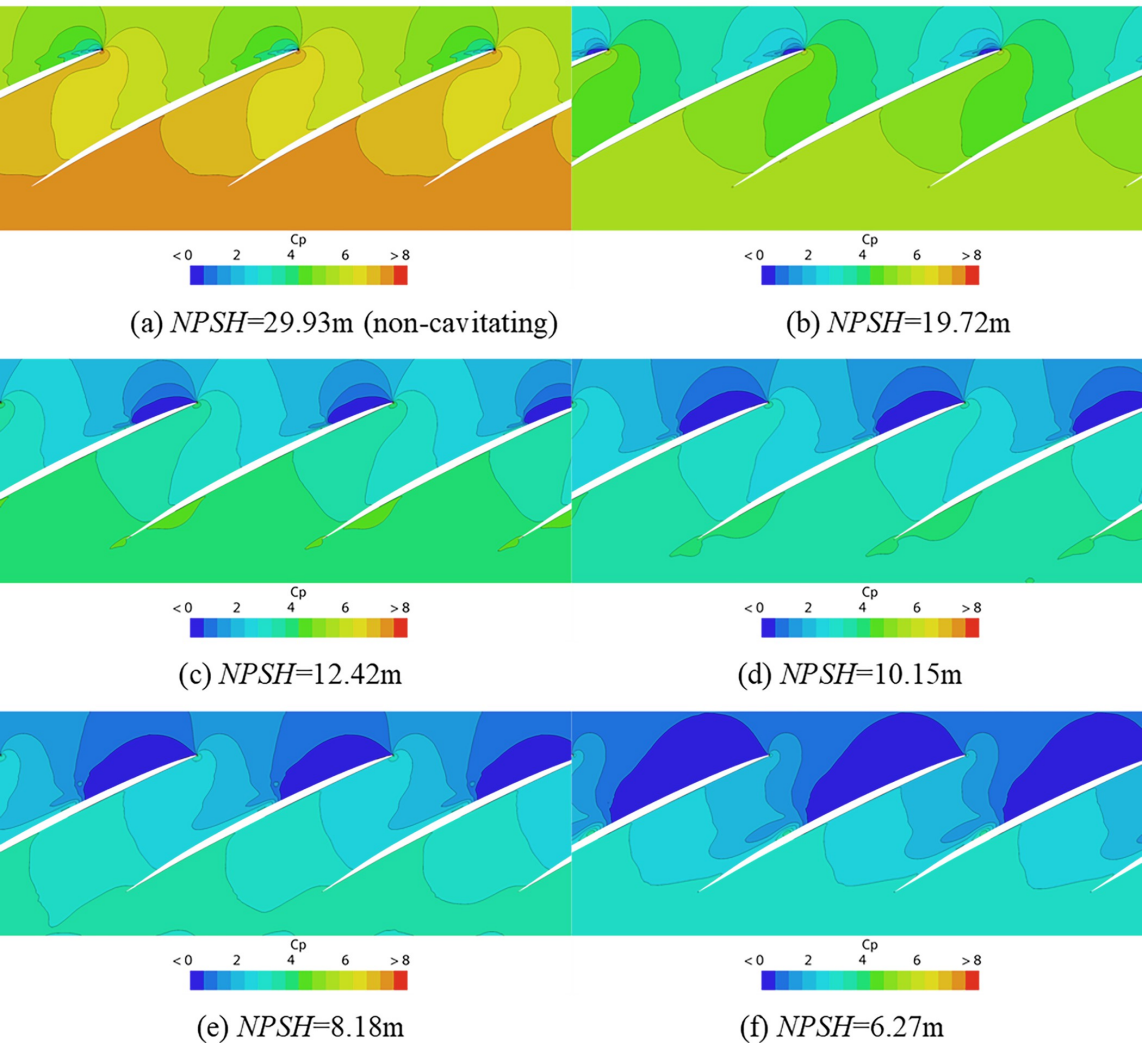
Pressure coefficient graph on cylindrical section at blade tip (expanding into plane).

The numerical simulation results are analyzed for the phenomenon of pulsating pressure variation with cavitation as shown in Fig 9. Fig 10 shows the distribution of pressure coefficient on three cross sections and blade surfaces, and the axial coordinates of these three cross sections are the same as those of the three monitoring points in the tip clearance. Fig 11 shows pressure coefficient graph on cylindrical section at blade tip (expanding into plane) whose radial coordinate is the same as that of the tip of the impeller. The region where the *C*_P_ is close to 0 in Figs 10 and 11 indicates that cavitation occurs here. As can be seen from Figs 10 and 11, the pressure pulsation is caused by the pressure difference between the suction side (low pressure area) and the pressure side (high pressure area) of the blade. The *C*_P_ on the blade tip in Fig 10 indicates that the pressure gradient is large at the downstream boundary of the cavitation region. For instance, at sensor7~10, before cavitation occurs here, the pressure on the suction side drops faster than that on the pressure side as cavitation intensifies. Therefore, the pressure difference between the two sides decreases, which is manifested as a decrease in pulsating pressure (*NPSH* = 29.92m to *NPSH* = 8.18m as shown in Fig 11). Once cavitation occurs here, the pressure on the suction side quickly drops to vaporization pressure, and the pressure difference between the two sides increases. Therefore, it is manifested as a rapid increase in pulsating pressure. With the further intensification of cavitation, the pressure on the suction side is still the vaporization pressure, while the pressure on the pressure side gradually decreases, which is manifested as the decrease of pulsating pressure.

## The similarity of tip clearance pulsating pressure

In order to investigate whether the variation of pulsating pressure in full scale waterjet propulsion pump is consistent with the model scale, the cavitation numerical simulation of the other two scale water-jet propulsion pumps is carried out, *D* = 0.6m and *D* = 0.9m, respectively. For different *D*, the size of the grid cell remains unchanged, and the *y*^+^ value remains about equal to 1, so the amount of grid increases significantly with the increase of *D*.

Firstly, the simulation conditions, *Q* and *N*_R_, of full scale water-jet propulsion pump should be determined according to the similarity of working conditions. The similarity of working conditions means that the flow coefficients (*C*_Q_) of different scale pumps are equal, and the *C*_Q_ is defined as,

$$C_{Q}=\frac{Q}{{(N}_{R}/60)D^{3}}$$ (7)

In the design condition of waterjet propulsion ship, Froude number ($Fr=V/\sqrt{Dg}$) similarity criterion is satisfied. Therefore, according to the equality of Froude numbers and$V=Q/(\frac{1}{4}\pi D^{2})$, the similarity criterion of *Q* can be obtained, i.e.,

$$\frac{Q_{f}}{Q_{m}}=\lambda^{2.5}$$ (8)

where the subscripts f and m denote model scale and full scale respectively; λ denotes scale ratio. According to the criterion that the *C*_Q_ of different scale waterjet propulsion pumps is equal when the operating conditions are similar, and Eq (7), Eq (8), the similarity criterion of *N*_R_ can be obtained, i.e.,

$$\frac{N_{Rf}}{N_{Rm}}=\lambda^{-0.5}$$ (9)

According to Eq (7), Eq (8) and Eq (9), the numerical simulation conditions of the other two scale pumps can be obtained, and the results are listed in Table 6.

**Table 6:** Numerical simulation conditions of different scale pumps.

*D*(m)	*λ*	*Q*(m^3^/s)	*N*_R_(r/min)	*C*_Q_
0.3	-	0.46	1450	0.705
0.6	2	2.6	1025.3	0.705
0.9	3	7.17	837.2	0.705

After determining the numerical simulation conditions, the numerical simulation of different cavitation conditions is carried out. Since the pulsating pressure near the leading edge is the largest, the variation of *RMS*_10_ of the pulsating pressure at the monitoring point near the leading edge with the variation of cavitation is investigated.

The range of *NPSH* varies greatly for different scale pumps. According to the definition of *NPSH*, Eq (2), a coefficient *C*_NPSH_ is defined in this study,

$$C_{NPSH}=\frac{NPSH}{V^{2}/g}=\frac{NPSH}{{\left(nD\right)}^{2}/g}$$ (10)

**Fig 12:**
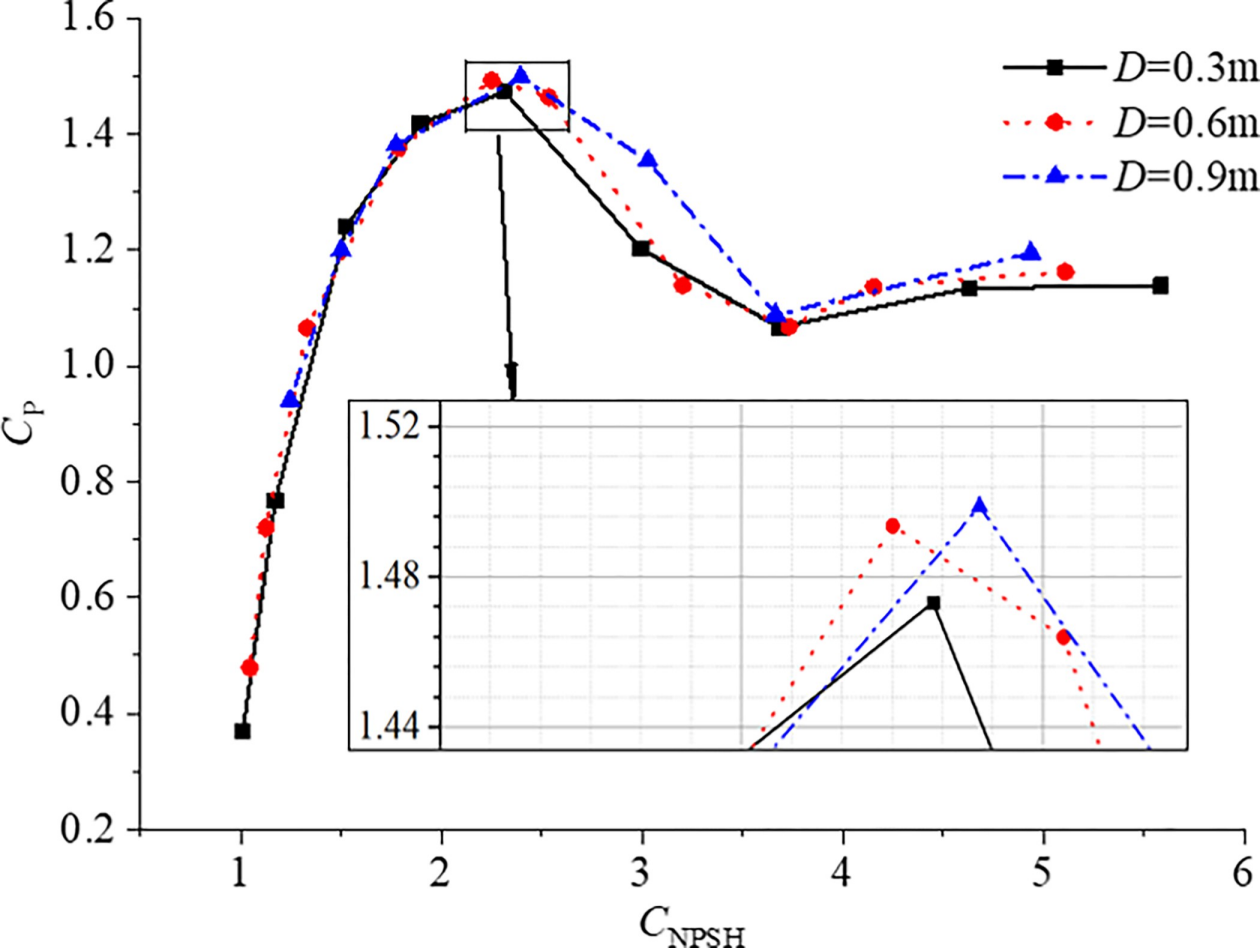
Variation of *RMS*_10_ of the pulsating pressure at the monitoring point near the leading edge.

The variation of *RMS*_10_ of the pulsating pressure at the monitoring point near the leading edge with the variation of cavitation is shown in Fig 12. The variation trend of *RMS*_10_ of the other two scale pumps is consistent with the conclusion analyzed according to Fig 9. The research shows that the maximum value of *RMS*_10_ increases slightly with the increase of scale due to the scale effect or numerical error. The cavitation pulsation pressure coefficients of pumps of different scales are equal when the working conditions are similar and the *C*_NPSH_ are equal, which can be used to predict the cavitation pulsating pressure performance of the full scale water-jet propulsion pump. The funding of this study is of great significance for engineering. However, the quantitative effects of scale effect need to be studied in more detail.

## Conclusion

The cavitation pulsating pressure in tip clearance of water-jet propulsion axial-flow pump is studied by means of experiments and RANS numerical simulation. Numerical uncertainty analysis is carried out to verify the feasibility of the numerical simulation method in this paper, and the main conclusions are as follows.

(1) The numerical uncertainty analysis shows that it is still difficult to simulate the cavitation pulsating pressure accurately, and the main errors are caused by numerical modeling, such as turbulence model and cavitation model.

(2) The experiments show that tip clearance cavitation starts from the leading edge, develops along the chord and towards the suction side with the decrease of *NPSH*. A stable cavitation region like a triangle is formed on suction side, and it breaks at a certain position, resulting in unstable small bubbles, and forms an unstable cavitation region. The unsteady RANS numerical simulation only simulates the stable cavitation region, which is one of the reasons for the error between numerical simulation and experiment.

(3) The *RMS*_10_ of the pulsating pressure (the total energy of the pulsating pressure) near the leading edge is the largest, in which the first-order blade frequency component is the main component. The numerical study found that the pulsating pressure at a certain position in the tip clearance decreases first, then increases, and then decreases with the intensification of cavitation.

(4) A dimensionless coefficient of *NPSH* is proposed in this paper. It is found that the maximum *RMS*_10_ and variation of cavitation pulsating pressure also satisfy the similarity criterion when the working conditions are similar and the *C*_NPSH_ are equal. However, due to the scale effect or maximum error, the *RMS*_10_ increases slightly as the scale increases. This means it is feasible to predict the cavitation pulsating pressure characteristics of full scale through the study of model scale, which is significant in practical engineering.

## Supporting information

S1 Table Comparison of *H* between simulation and experiment. (PDF)

S2 Table First 10 blade frequency components of pulsating pressure in the tip clearance with different *NPSH*. (PDF)

S3 Table Variation of *RMS*_10_ of the pulsating pressure at the monitoring point near the leading edge. (PDF)
