# Supplementary material for: Experimental and numerical study on cavitation pulsating pressure of water-jet propulsion axial-flow pump
Source: PLoS One. 2024 Oct 28;19(10):e0310167. doi: 10.1371/journal.pone.0310167 (PMC11516003; doi:10.1371/journal.pone.0310167)
Supplement: S1 Table — (PDF) [file pone.0310167.s001.pdf]

S1 table. Comparison of *H* between simulation and experiment

NPSH(m)	D_E(m)	D_S(m)	|E|(%)
15.2	--	13.13	--
12.42	12.73	13.127	3.1
12.2	12.74	13.124	3.0
10.15	12.65	13.087	3.5
8.18	12.56	13.0154	3.6
6.27	11.9	12.337	3.7
