# Supplementary material for: Experimental and numerical study on cavitation pulsating pressure of water-jet propulsion axial-flow pump
Source: PLoS One. 2024 Oct 28;19(10):e0310167. doi: 10.1371/journal.pone.0310167 (PMC11516003; doi:10.1371/journal.pone.0310167)
Supplement: S2 Table — (PDF) [file pone.0310167.s002.pdf]

S2 table. First 10 blade frequency components of pulsating pressure in the tip clearance with different *NPSH*

(a) *NPSH*=12.42m	D_S		
Multiple of blade frequency	C_P:sensor2	C_P:Sensor7~10	C_P:Sensor3
1	1.07666	0.43175	0.14468
2	0.77928	0.1953	0.05651
3	0.47772	0.12631	0.03879
4	0.23627	0.09362	0.02903
5	0.11364	0.07062	0.02338
6	0.13458	0.05846	0.01876
7	0.17054	0.04853	0.0159
8	0.17043	0.04408	0.01291
9	0.13254	0.03862	0.01007
10	0.07129	0.03675	0.00989
(a) *NPSH*=12.42m	D_E		
Multiple of blade frequency	C_P:sensor2	C_P:Sensor7~10	C_P:Sensor3
1	1.25459	0.41068	0.14613
2	0.71976	0.20755	0.05565
3	0.13218	0.13789	0.04011
4	0.21881	0.09979	0.03657
5	0.2422	0.08376	0.02965
6	0.09868	0.07237	0.02169
7	0.16914	0.06505	0.01868
8	0.06953	0.05532	0.01251
9	0.03285	0.04466	0.00903
10	0.05073	0.03052	0.00713

(b) *NPSH*=10.15m	D_S		
Multiple of blade frequency	C_P:sensor2	C_P:Sensor7~10	C_P:Sensor3
1	1.19796	0.36153	0.12805
2	0.61814	0.14951	0.05932
3	0.22767	0.09006	0.04152
4	0.20311	0.06793	0.03242
5	0.1842	0.05356	0.02526
6	0.15066	0.04787	0.0209
7	0.10939	0.03811	0.01793
8	0.09912	0.0336	0.01484
9	0.10108	0.02838	0.01177
10	0.08505	0.02729	0.01119
(b) *NPSH*=10.15m	D_E		
Multiple of blade frequency	C_P:sensor2	C_P:Sensor7~10	C_P:Sensor3
1	1.3391	0.3113	0.13252
2	0.35949	0.12606	0.05145
3	0.28077	0.07684	0.03335
4	0.2535	0.04831	0.0213
5	0.18304	0.04188	0.02333
6	0.24629	0.04221	0.02224
7	0.23437	0.041	0.02003
8	0.13245	0.03077	0.01604
9	0.01865	0.02289	0.01278
10	0.07872	0.01636	0.00972

(c) *NPSH*=8.18m	D_S		
Multiple of blade frequency	C_P:sensor2	C_P:Sensor7~10	C_P:Sensor3
1	1.10953	0.34435	0.12099
2	0.38114	0.12287	0.05592
3	0.25292	0.03682	0.04084
4	0.17777	0.014	0.03171
5	0.13916	0.02555	0.02554
6	0.12457	0.03171	0.0213
7	0.09415	0.02806	0.01909
8	0.09339	0.02525	0.01605
9	0.08338	0.01833	0.01321
10	0.06318	0.01489	0.01271
(c) *NPSH*=8.18m	D_E		
Multiple of blade frequency	C_P:sensor2	C_P:Sensor7~10	C_P:Sensor3
1	1.12497	0.29252	0.10741
2	0.2532	0.06504	0.05277
3	0.24222	0.00942	0.02459
4	0.20495	0.03386	0.03195
5	0.27752	0.05365	0.0316
6	0.22819	0.05984	0.03206
7	0.26355	0.05035	0.02363
8	0.10544	0.03383	0.01725
9	0.03419	0.02059	0.01153
10	0.09075	0.01614	0.00981

(d) *NPSH*=6.27m	D_S		
Multiple of blade frequency	C_P:sensor2	C_P:Sensor7~10	C_P:Sensor3
1	0.65686	0.54041	0.17798
2	0.2844	0.33049	0.05298
3	0.16613	0.26748	0.02155
4	0.12737	0.28513	0.01705
5	0.09223	0.26465	0.01503
6	0.07921	0.20824	0.01144
7	0.07415	0.13953	0.01252
8	0.06443	0.09242	0.01214
9	0.05704	0.07578	0.01154
10	0.04983	0.07522	0.01018
(d) *NPSH*=6.27m	D_E		
Multiple of blade frequency	C_P:sensor2	C_P:Sensor7~10	C_P:Sensor3
1	0.5515	0.92469	0.20112
2	0.22845	0.25301	0.1138
3	0.17007	0.11207	0.05443
4	0.21564	0.11085	0.0637
5	0.18803	0.08915	0.04726
6	0.1275	0.02169	0.04203
7	0.07476	0.02862	0.03705
8	0.03625	0.01544	0.02529
9	0.01403	0.01059	0.01369
10	0.01732	0.00288	0.00203
