# Supplementary material for: Experimental and numerical study on cavitation pulsating pressure of water-jet propulsion axial-flow pump
Source: PLoS One. 2024 Oct 28;19(10):e0310167. doi: 10.1371/journal.pone.0310167 (PMC11516003; doi:10.1371/journal.pone.0310167)
Supplement: S3 Table — (PDF) [file pone.0310167.s003.pdf]

S3 table. Variation of RMS_{10} of the pulsating pressure at the monitoring point near the leading edge

D=0.3m		D=0.6m		D=0.9m	
C_{NPSH}	C_P	C_{NPSH}	C_P	C_{NPSH}	C_P
5.5859	1.13947	5.1086	1.16286	4.93717	1.19522
4.63228	1.13478	4.15454	1.13738	3.66416	1.08825
3.6804	1.06732	3.73343	1.06855	3.02917	1.35411
2.99456	1.20237	3.2008	1.13959	2.39537	1.49876
2.318	1.47313	2.5347	1.46394	1.77283	1.38167
1.8945	1.41894	2.25122	1.49354	1.49943	1.2002
1.5263	1.24131	1.78529	1.37587	1.24533	0.94133
1.17022	0.7671	1.32963	1.06586		
1.00895	0.369	1.12533	0.72103		
		1.04503	0.47857		
